# Global Trends in Research of Mitochondrial Biogenesis over past 20 Years: A Bibliometric Analysis

**DOI:** 10.1155/2023/7291284

**Published:** 2023-01-04

**Authors:** Lei Song, Jiaqi Liang, Wenting Wang, Jie Gao, Hua Chai, Yu Tan, Liying Zheng, Mei Xue, Dazhuo Shi

**Affiliations:** ^1^Center of Cardiovascular Disease, Xiyuan Hospital, China Academy of Chinese Medical Sciences, 100091 Beijing, China; ^2^Graduate School, Beijing University of Chinese Medicine, 100029 Beijing, China; ^3^National Clinical Research Center for Chinese Medicine Cardiology, Xiyuan Hospital, China Academy of Chinese Medical Sciences, 100091 Beijing, China; ^4^Xiyuan Hospital, China Academy of Chinese Medical Sciences, 100091 Beijing, China; ^5^Department of Cardiovascular Biology and Medicine, Juntendo University Graduate School of Medicine, 113-8421 Tokyo, Japan; ^6^Department of Cardiovascular Disease, Hangzhou Red Cross Hospital, 310003 Hangzhou, China; ^7^Department of Geriatrics, Hepingli Hospital, 100013 Beijing, China; ^8^Graduate School, China Academy of Chinese Medical Sciences, 100700 Beijing, China

## Abstract

**Background:**

Mitochondrial biogenesis-related studies have increased rapidly within the last 20 years, whereas there has been no bibliometric analysis on this topic to reveal relevant progress and development trends.

**Objectives:**

In this study, a bibliometric approach was adopted to summarize and analyze the published literature in this field of mitochondrial biogenesis over the past 20 years to reveal the major countries/regions, institutions and authors, core literature and journal, research hotspots and frontiers in this field.

**Methods:**

The Web of Science Core Collection database was used for literature retrieval and dataset export. The CiteSpace and VOSviewer visual mapping software were used to explore research collaboration between countries/regions, institutions and authors, distribution of subject categories, core journals, research hotspots, and frontiers in this field.

**Results:**

In the last 20 years, the annual number of publications has shown an increasing trend yearly. The USA, China, and South Korea have achieved fruitful research results in this field, among which Duke University and Chinese Academy of Sciences are the main research institutions. Rick G Schnellmann, Claude A Piantadosi, and Hagir B Suliman are the top three authors in terms of number of publications, while RC Scarpulla, ZD Wu, and P Puigserver are the top three authors in terms of cocitation frequency. PLOS One, Biochemical and Biophysical Research Communications, and Journal of Biological Chemistry are the top three journals in terms of number of articles published. Three papers published by Richard C Scarpulla have advanced this field and are important literature for understanding the field. Mechanistic studies on mitochondrial biosynthesis have been a long-standing hot topic; the main keywords include skeletal muscle, oxidative stress, gene expression, activation, and nitric oxide, and autophagy and apoptosis have been important research directions in recent years.

**Conclusion:**

These results summarize the major research findings in the field of mitochondrial biogenesis over the past 20 years in various aspects, highlighting the major research hotspots and possible future research directions and helping researchers to quickly grasp the overview of the developments in this field.

## 1. Introduction

Mitochondria play key roles in the energy supply, signaling, and apoptosis of cells [1]. The normal functions of mitochondria depend on the coordination of mitochondrial biogenesis, mitochondrial dynamics, and mitophagy [2]. A series of studies have shown that mitochondrial dysfunction is associated with numerous human conditions, such as cancer [3], metabolic diseases [4], neurodegeneration [5], diabetes [6], and aging [7]. Through biogenesis, mitochondria generate new mitochondria through replication and division. Mitochondrial biogenesis intervention and regulatory transcriptional network alterations are important methods of regulating mitochondrial function [8]. Mitochondrial biogenesis is a complex biological process that requires encoding of both mitochondrial-nuclear DNA and mitochondrial DNA. The precise coordination between nuclear DNA and mitochondrial-DNA transcription ensures mitochondrial adaptation to various physiological and pathological environmental changes and performance of normal functions [9, 10]. Peroxisome proliferator-activated receptor gamma coactivator-1*α* (PPARGC1A/PGC-1*α*), nuclear respiratory factors (NRF1 and NFE2L2/NRF2), and transcription factor A, mitochondrial (TFAM) are major players in mitochondrial biogenesis regulation [11, 12], and a series of factors can affect mitochondrial biogenesis by regulating the expression or posttranslational modifications of PGC-1*α* [13, 14]. Mitochondrial biogenesis regulates the abundance and functional properties of mitochondria and has attracted widespread attention, with the number of relevant publications increasing every year. Therefore, it is necessary to summarize the published literature to clarify the development conditions, research hotspots, and frontiers in the mitochondrial biogenesis field.

Bibliometrics is a research method in which all knowledge carriers are objectively and quantitatively analyzed through mathematical and statistical methods [15]. It is often used to analyze academic publications in a certain field to clarify overall trends in a field and assess the relative importance of academic achievements. Recently, bibliometric methods have been widely used in various research fields including Clinical Medicine [16, 17], Biology [18, 19], Sociology [20, 21], Education [22], and Science and Engineering [23, 24] and have played important roles in helping researchers quickly grasp research hotspots and trends in a specific field. The CiteSpace software was developed with Java programming language by Prof. Chaomei Chen of Drexel University [25]. Through data mining, data analysis and visualization, CiteSpace illustrates the structure, patterns, and distribution of scientific knowledge. Collaborative network analysis, cooccurrence analysis, and cocitation analysis can be carried out with CiteSpace. The VOSviewer software, developed by the Center for Scientific and Technological Research (Department of Bibliometrics) at Leiden University, Netherlands, provides three types of visual maps: network, label, and density maps from which to generate visual clusters of different objects [26].

There are no published papers analyzed by bibliometric methods in the field of mitochondrial biogenesis. Therefore, this study analyzes the published literature in the field of mitochondrial biogenesis over the past 20 years by using CiteSpace and VOSviewer visual mapping software to reveal global collaborative network relationships and identify key researchers, core literature, research hotspots, and frontiers in this field. The results of these analyses will help future researchers to quickly grasp the overall research progress in the field.

## 2. Methods

### 2.1. Data Acquisition and Search Strategy

The Web of Science (WoS, Clarivate Analytics, Philadelphia, PA, USA) database is a collection of more than 12,000 international academic journals and is one of the most comprehensive and authoritative database platforms for accessing global academic resources [27]. In addition, it features a citation index search, which is a necessary tool for bibliometric cocitation analysis [28]. Therefore, the WoS database was selected as the data source for this study.

**Figure 1:**
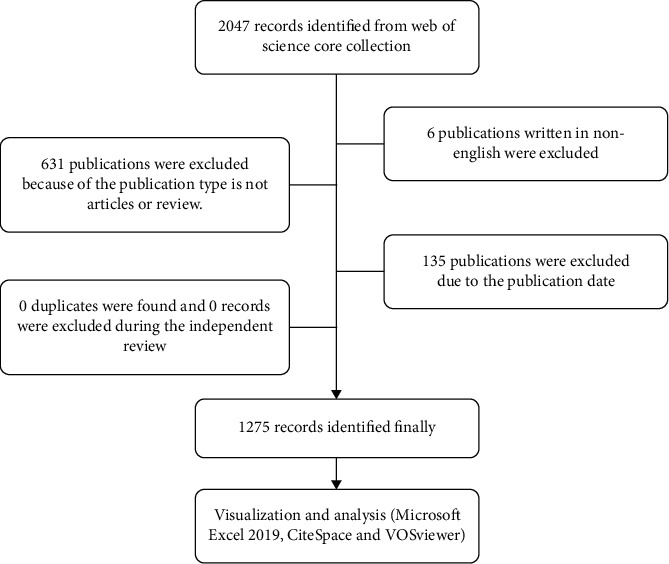
Flowchart of the literature screening.

All relevant literature was retrieved and exported from the Web of Science Core Collection (WoSCC) database with the search formulas Title = (^“^mitochondria^∗^ biogenesis^”^) and Title = (^“^mitochondria^∗^ biosynthesis^”^). The preliminary search yielded 2047 records, and a total of 1275 of these records were included in the data analysis set based on the results refinement function in the WoSCC database, which was used with the following settings: language, English; document types, articles and review articles; publication dates, from January 1, 2002 to December 31, 2021, (Figure 1). A total of 1275 records were selected as “full record with cited references,” exported into “plain text file format,” and renamed with the “download^∗^.txt” convention to ensure that they were read correctly by the CiteSpace and VOSviewer software.

### 2.2. Data Extraction

A total of 1275 documents were imported into CiteSpace (5.8.R3) software, and duplicates were thus quickly removed. The acquired documents were then manually reviewed by two independent researchers to ensure article relevance to mitochondrial biogenesis. When these researchers disputed the relevance of a paper, they read the original article together and reached an agreement.

### 2.3. Data Analysis

Microsoft Excel 2019 software (Microsoft Corporation, Redmond, WA, USA) was used to prepare graphs showing the number of publications per year. CiteSpace software was used to perform country, institution, and author cooperation network analyses, a subject category analysis, a reference cocitation analysis, and a keyword cooccurrence analysis, and VOSviewer software was used to carry out journal cooccurrence and cocitation analyses.

The size of the nodes is positively correlated with the frequency of the cooccurrence or cocitation of analyzed objects. The lines between the nodes indicate a cooccurrence or cocitation relationship, and the thickness of the lines indicates the strength of the relationship between objects. The color of the colored rings and lines around a node indicate the year in which the object or relationship first appeared in the literature. The purple circles around certain nodes indicate betweenness centrality (BC), which shows the importance of a node within the whole network. Nodes with BC > 0.1 are marked with purple rings. The thickness of a purple ring is proportional to the BC value. Nodes with usage bursts are visualized by red rings, which indicate that the study object represented by the node appeared at a high frequency in a certain period [29, 30].

VOSviewer software distinguishes different clusters by different colors [31]. The size of different colored circles is proportional to the number of different analyzed objects, the distance between different circles is inversely proportional to the number of objects, and the thickness of connecting lines reflects the strength of the interrelationship between the connected objects [32].

## 3. Results

### 3.1. Trends in Publications on Mitochondrial Biogenesis

**Figure 2:**
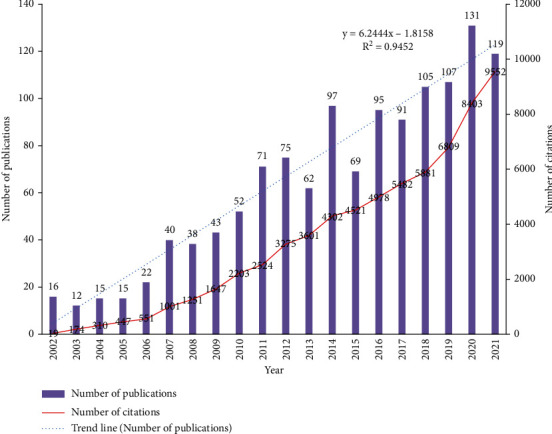
Annual publication output and citations between 2002 and 2021.

Between 2002 and 2021, a total of 1275 articles referring to mitochondrial biogenesis in the title were retrieved from the WoSCC database.  Figure 2 showed that the annual publication volume and citations of mitochondrial biogenesis-related papers [33]. An overall steady upward trend is observed, although a surge and a market drop were evident in 2014, indicating that in the past 20 years, mitochondrial biogenesis has gradually attracted the attention of researchers. The type of mitochondrial biogenesis-related papers was mainly article (*n* = 1166) and review (*n* = 109), indicating that the mitochondrial biogenesis field has been dominated by original research, and that, a summary analysis of the relevant original research published in this field is needed.

### 3.2. Country/Region and Institution Cooperation Network Analyses

**Figure 3:**
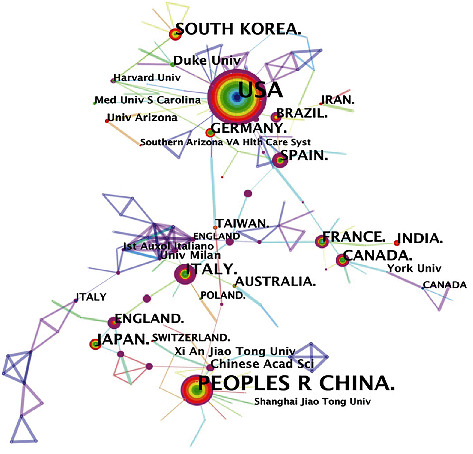
The country/region and institution cooperation network analyses.

Researchers from 69 countries and 292 institutions participated in scientific research into mitochondrial biogenesis. The United States presented the highest number of collaborative publications (462), and Duke University in the United States was credited with the most collaborative publications in this field (30). Mainland China ranked 2nd (290), but the number of collaborative publications was only approximately 1/2 that of the United States (Table 1). The Chinese Academy of Sciences was the main research institution in China, and it ranked 2nd (23) among global institutions. In addition, both the United States and China were credited with a high publication number and high betweenness centrality (BC) values, suggesting that these countries have shown high proliferation of both quantitative and qualitative research in this field. The countries that followed in terms of the number of cooperative publications (in parentheses) are South Korea (69), Italy (62), Japan (55), Spain (51), and Canada (48). In addition, the BC values attributed to Japan, Spain, France, and Germany were all >0.1, suggesting that these countries are highly involved in national collaborative networks.  Figure 3 shows significantly more cooperative links between institutions than between countries, suggesting that the cooperative relationship between major research institutions in each country is high. However, the BC value of the number of cooperative documents for the top ten cooperative institutions is not high, indicating that the interaction and cooperation between institutions in different countries are relatively low. Notably, researchers in the United States carried out mitochondrial biogenesis-related research approximately 5 years earlier than those in other countries, as indicated by the year of the first publication.

### 3.3. Author Cooperation and Cocitation Network Analyses

**Figure 4:**
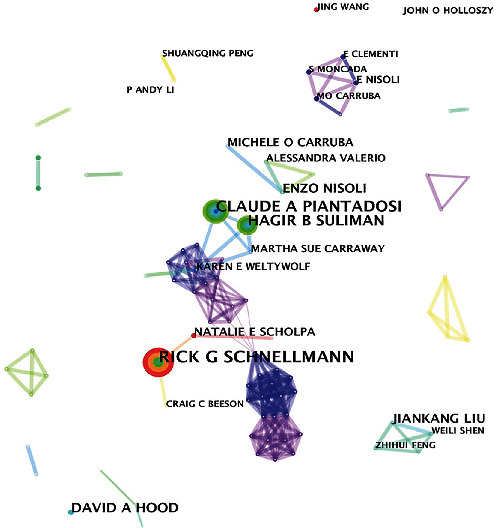
Author cooperation network analysis.

**Figure 5:**
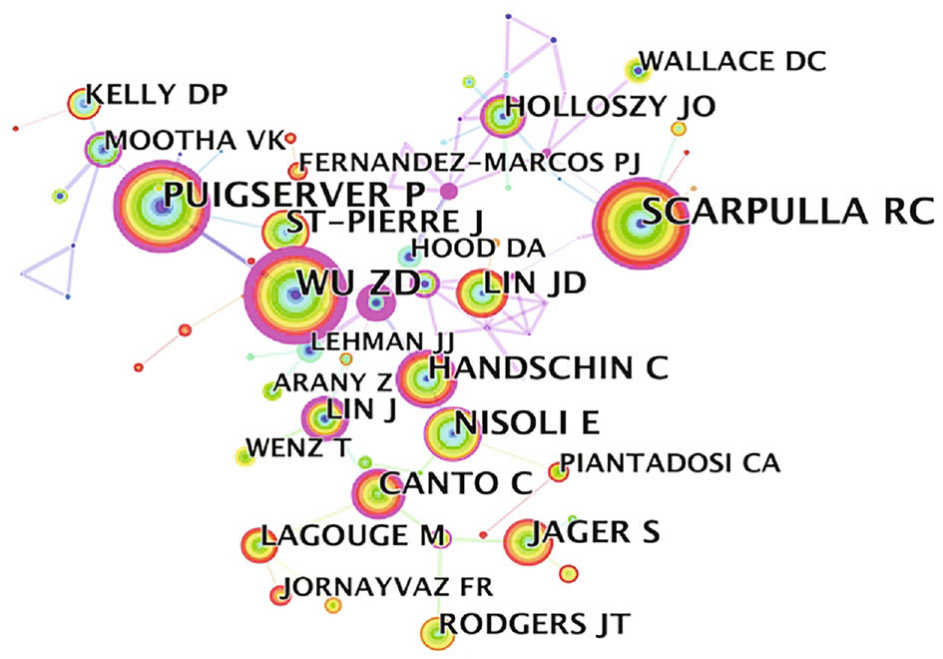
Author cocitation network analysis.

A total of 618 authors participated in the field of mitochondrial biogenesis. As shown in Table 2, the three most proliferative authors in terms of the number of collaborative publications were Rick G Schnellmann, Claude A Piantadosi, and Hagir B Suliman; however, none of the top ten authors exhibited a BC value greater than 0.1, suggesting that the correlation between the research topics and these authors is not high. As shown in Figures 4 and 5, each node represents an author, the links between circles represent the connections between authors, and different link colors represent different publication periods. An author cocitation indicates that two or more authors are cited by other papers in the same period, and therefore, these authors exhibit a cocitation relationship. The top five cocited authors were RC Scarpulla, ZD Wu, P Puigserver, E Nisoli, and C Handschin, and the BC values of these authors were all >0.1, indicating that their research results have played an important role in promoting the development of the mitochondrial biogenesis field.

### 3.4. Subject Category Cooccurrence Analysis

**Figure 6:**
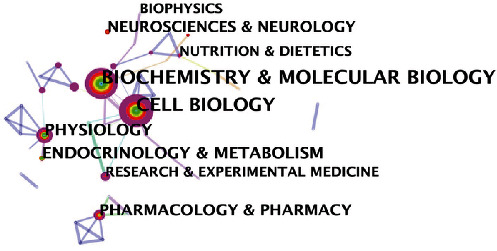
Subject categories in the cooccurrence network analysis.

By carrying out a cooccurrence analysis between disciplines, the related disciplines in the mitochondrial biogenesis field can be identified.  Table 3 shows that mitochondrial biogenesis is a relatively concentrated subject. Biochemistry and Molecular Biology, Cell Biology, and Physiology are the categories with most of the published literature on mitochondrial biogenesis. These subjects are in the field of biology, and all have high BC values, which are indicated by purple circles in Figure 6. In addition, the most relevant mitochondrial biogenesis topics included Endocrinology and Metabolism, Neurosciences and Neurology, and Pharmacology and Pharmacy in the field of medicine, all of which exhibited a cooccurrence frequency greater than 100-fold. Among these topics, Pharmacology and Pharmacy are shown in red circles, indicating that this subject was a hot topic in a certain period.

### 3.5. Journal Cooccurrence and Cocitation Analyses

**Figure 7:**
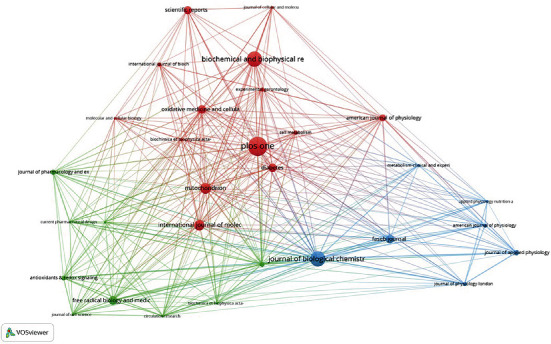
Journal cooccurrence network analysis.

**Figure 8:**
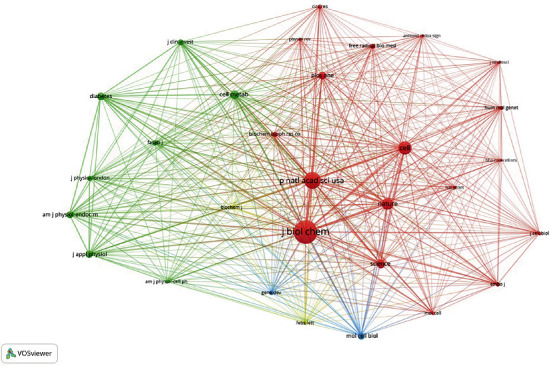
Journal cocitation network analysis.

VOSviewer software was used to analyze which journals mainly published literature related to mitochondrial biosynthesis (Figures 7 and 8). Among them, PLOS One published the most papers (38), followed by Biochemical and Biophysical Research Communications (30), and Journal of Biological Chemistry (30). Among the top 10 journals in terms of publication count, Diabetes had the highest impact factor (IF), which was 9.461. Furthermore, 80% of the journals are in the first quartile (Q1) or Q2. The number of citations may reflect the influence of the journal. The journal with the highest number of citations was the Journal of Biological Chemistry, followed by Proceedings of the National Academy of Sciences of the United States of America. According to the 2020 Journal Citation Reports (JCR), 100% of the top journals in this analysis was in Q1 or Q2 (Table 4).

### 3.6. Reference Cocitation Analysis

The 3 most cocited papers were all published by Richard C Scarpulla, indicating that his research results have greatly promoted the development of this field of mitochondrial biogenesis (Table 5). He is a professor of Cell and Developmental Biology at Northwestern University. His research topics include medicine and life sciences and chemical compounds. His main research publications involve nuclear respiratory factor 1, cytochrome C, organelle biogenesis, mitochondrial genes, electron transport, etc. Scarpulla's manuscripts introduced related pathways and protein regulatory networks that mediate mitochondrial biogenesis in detail, which help other researchers better understand mitochondrial biogenesis.

### 3.7. Keyword Cooccurrence Analysis

**Figure 9:**
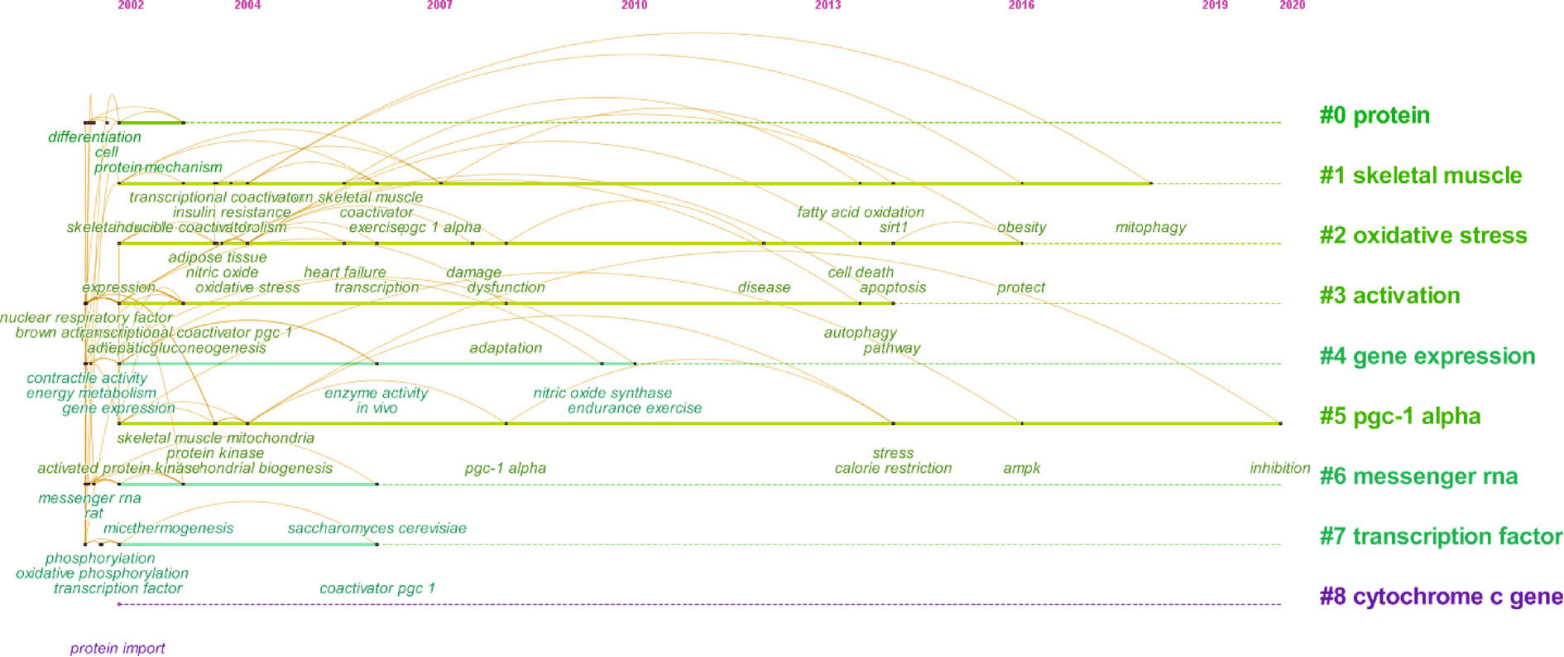
The diagram showing keyword clustering and timeline analysis.

**Figure 10:**
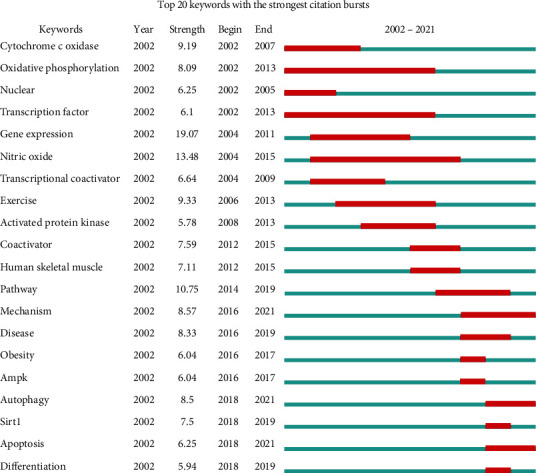
The 20 most cited keywords as determined by a keyword burst analysis ordered by occurrence year.

As shown in Table 6, compared with the keywords from the 11th to the 20th position, the keywords from the 1st to the 10th position not only showed a higher cooccurrence frequency but also exhibited a higher BC value, suggesting that the top 10 keywords were the most important in the mitochondrial biogenesis field and can best reflect the research hotspots in the field in a specific period. The top 10 keywords can be roughly classified into three main categories: the main research object, genes or proteins related to mitochondrial biogenesis regulation, and biological processes related to mitochondrial biogenesis. In addition, the relationship between keywords was explored by the keyword clustering timeline graph generated by CiteSpace (Figure 9). The clustering results revealed the cluster labels of the most frequently cited keywords. The smaller the value of the cluster label was, the more members there were in the cluster. Modularity *Q* = 0.7086 and Silhouette *S* = 0.9049 indicated that the clustering structure was appropriate and that the clustering results were very credible; that is, the keyword cluster labels are consistent with the meaning of the top 10 keywords. In addition, a keyword burst analysis was carried out to show changes in keyword frequency over time, thereby highlighting the changing trend of research hotspots in the mitochondrial biogenesis field. The result showed that in 2002, researcher attention on mitochondrial biogenesis was mainly focused on mitochondrial processes in energy metabolism, primary processes involved with oxidative phosphorylation, and that this research hotspot persisted for approximately 10 years, mainly involving the regulation of transcription and translation and expression of genes and proteins related to mitochondrial biogenesis, including PGC-1. Mechanistic research related to mitochondrial biogenesis has become a hot spot in the past 5 years, mainly involving through in vitro experiments or clinical trials to explore whether a certain intervention can play a therapeutic role by regulating mitochondrial biogenesis (Figure 10).

## 4. Discussion

### 4.1. General Information

Based on the method of bibliometric, CiteSpace and VOSviewer software were used to analyze papers related to the field of mitochondrial biogenesis published in the WoSCC database for the last 20 years in the expectation of revealing the progress trends, research hotspots, and research frontiers in this field. Within the study period, mitochondrial biogenesis has received extensive attention from researchers around the world. The number of papers published on mitochondrial biogenesis has followed an overall upward trend every year, and the type of literature has been mainly original articles. Duke University in the United States and the Chinese Academy of Sciences in China have been the main research institutions publishing articles in this field. Researchers from North America, Asia, and Europe have led mitochondrial biogenesis-related research, with the United States and China have shown a leading trend in the number of cooperative publications. However, although China ranks second in terms of the number of publications, it does not show corresponding advantages in author cocitation or reference cocitation rankings, suggesting that research results from China playing a leading role in this field are lacking. In addition, the cooperation between authors in this field is not strong. Rick G Schnellmann has produced the most publications and has been cocited at the highest frequency. He has published a series of review articles on the mitochondrial biogenesis transcriptional regulatory network, which has greatly promoted development of this field. The disciplines involved in the mitochondrial biogenesis field are concentrated in the major categories of biology, mainly cell biology, molecular biology, biochemistry, and other subdisciplines. The development of basic research has also promoted the transformation and application of relevant research results to clinical medicine and pharmacy. Most journals that publish papers related to mitochondrial biogenesis topics are Q1 or Q2 journals. Notably, highly recognized high-level journals such as Nature [39], Science [40], and Cell [41] were ranked high in the journal cocitation analysis (from 6th to 8th), suggesting that this field has attracted extensive attention from the academic community.

### 4.2. The Analysis of Top 10 Cocited Literatures

The top 4 cocited articles were reviews, which published by Richard C Scarpulla in 2004, 2008, 2011, and 2012 as the first author or corresponding author. These papers discussed in detail the mitochondrial biosynthesis of PGC-1 coactivators and belonged to JCR Q1 in the year of publication, indicating that the research results of these reviews contributed to the promotion of the same field's researchers to understand the biological mechanism of mitochondrial biosynthesis and had become classic literatures in this research field. The documents ranked 5th and 6th revealed the main upstream regulation mechanism of PGC-1*α* based on experimental research, mainly including the activation of AMP-activated kinase which can be directly phosphorylated by direct phosphorylation on two critical residues, threonine-177 and serine-538, which promote the expression of PGC-1*α* in skeletal muscle; in addition, by increasing cellular NAD+ levels to enhance the activity of SIRT1, resulting in the deacetylation and modulation of the activity of downstream SIRT1 targets and triggers PGC-1*α* deacetylation, thereby, plays a role in the intervention and regulation of mitochondrial energy metabolism. The 7th-ranked document proposed that endogenous nitric oxide can also regulate mitochondrial biosynthesis through guanosine 3,5-monophosphate-dependent manner. This discovery may provide a new method for the prevention and treatment of mitochondrial dysfunction diseases in the clinical practice. The 8th-ranked document showed that PGC-1*α* has a bidirectional regulatory function, which can stimulate mitochondrial electron transport and suppress ROS levels to keep the body in a relative balance state between energy metabolism requirements and oxidative stress damage, which provided new insights into understanding the physiological functions of PGC-1*α*. The 9th-ranked document is also a review, which summarized and analyzed the mechanism of host cell dynamic regulation of oxidative stress-mediated mitochondrial damage and mitochondrial biosynthesis. The 10th-ranked literature showed through experimental studies that resveratrol induces PGC-1*α* activity by facilitating SIRT1-mediated deacetylation, which demonstrated the potential of natural products for regulating mitochondrial biosynthesis. Collectively, these articles mapped the biological regulatory network associated with mitochondrial biosynthesis revealed the important role of the PGC-1*α* family and its upstream and downstream influencing factors. These explained why they had been widely cited for providing a theoretical reference for subsequent application-based studies on diagnostic mitochondrial biosynthesis and forming a cornerstone for further development of the field.

### 4.3. Research Hotspots and Frontiers

Based on the results of keyword cooccurrence analysis and cluster analysis, the research hotspots in the field of mitochondrial biogenesis can be reasonably inferred. Research in the past ten years has focused mainly on the regulatory network of mitochondrial biogenesis in skeletal muscle, such as study into PGC-1*α*/NRF/TFAM, the core pathway in mitochondrial biogenesis, and the influence of various factors on this pathway [44, 45].

#### 4.3.1. Skeletal Muscle

The biological process of mitochondrial biogenesis was first discovered through a comparison of exercised and nonexercised muscle tissue samples [46]. In 1960, Holloszy suggested that the increased mitochondrial electron transport observed in muscle tissue may be caused by an increase in a then-unknown process, mitochondrial biogenesis. Mitochondrial biogenesis increases the number of mitochondria and expression of ATP and promotes the aerobic metabolism of muscle tissue [47]. Skeletal muscle cells are among the cell types with the most vigorous energy metabolism and require a constant supply of energy to maintain physical activity [48]. Therefore, many mitochondrial biogenesis-related studies have focused on skeletal muscle as the main research object [49–51]. In addition, because of their high-energy dependence, cardiomyocytes and neuronal cells have become common research objects in mitochondrial biogenesis studies [52, 53]. Many studies have shown that endurance training can improve exercise tolerance [54–56], and the mechanism of this tolerance is mainly manifested in the transformation of skeletal muscle fiber types [57], increase in capillaries, and enhancement of mitochondrial biogenesis [58–60]. The enhancement of mitochondrial biogenesis is fundamentally important as it leads to greater rates of oxidative phosphorylation and an improved ability to utilize fatty acid oxidation. A series of studies have shown that this higher efficiency may be related to reduced oxidative stress and promoted AMP-activated protein kinase (AMPK) expression [61, 62]. AMPK is an intracellular energy sensor that can detect the energy status of skeletal muscle cells. When the intracellular ATP/AMP ratio decreases, AMPK is activated and then participates in the initiation of mitochondrial biogenesis [63, 64], which increases the fatty acid uptake by skeletal muscle cells, increasing the lipid oxidation rate and the transport of fatty acids into mitochondria, thereby increasing ATP production [65].

#### 4.3.2. Biological Processes, Gene Expression, and Regulatory Transcriptional Networks in Mitochondrial Biogenesis

In mitochondrial biogenesis, mitochondria generate new mitochondria by fission and self-replication, which includes transcription and translation of mitochondrial and nuclear DNA, recruitment of specific factors to mitochondrial membranes, protein import in mitochondria, and oxidative phosphorylation complex assembly. The proteins required for mitochondrial biogenesis are encoded by both nuclear and mitochondrial genes [66], with the mitochondrial genome encoding only 13 synthesized proteins [67]. Studies have confirmed that PGC-1*α* is the main regulator of mitochondrial biogenesis and is abundant in tissues with high oxidative activity, such as heart, brown adipose tissue, skeletal muscle, and brain tissue [68–70]. It can activate nuclear respiratory factors, increase nuclear transcription of the mitochondrial genome, activate mitochondrial transcription factors, and promote the transcription and replication of mitochondrial DNA. In addition, PGC-1*α* can coactivate other transcription factors, such as peroxisome proliferator-activated receptor (PPAR), estrogen, and estrogen-related factor (ERR) *α* and *γ* receptors [71], to regulate different aspects of energy metabolism, including mitochondrial biogenesis, fatty acid oxidation, and antioxidative processes. Mitochondrial biogenesis involves a complex regulatory transcriptional network, and PGC-1*α* activity triggered by external physiological stimuli is involved in the functional regulation of mitochondrial biogenesis by coactivating and controlling the expression of this transcription network. Many studies have shown that the gene expression or activity of PGC-1*α* is regulated by many factors, such as sirtuins (SIRTs), AMPK, p38 mitogen-activated protein kinase (p38 MAPK), thyroid hormone, *β*-adrenergic stimulation, nitric oxide synthase (NOS/cGMP), calcineurin, and calmodulin-activated kinases (CaMKs) [72–76].

#### 4.3.3. Oxidative Stress and Apoptosis

Reactive oxygen species (ROS) are natural byproducts of normal oxygen metabolism in the body, and they regulate intracellular signaling and play important roles in many physiological and pathological conditions [77]. However, abnormal and dramatic increases in ROS levels can cause cell death through apoptotic or necrotic pathways in a process known as oxidative stress [78, 79]. Therefore, normal ROS levels need to be maintained in an organism. The main function of mitochondria is to supply energy to a cell by generating ATP through oxidative phosphorylation. In addition, mitochondria are the main organelles involved in intracellular ROS production and the regulation of apoptosis and have received much attention from researchers in recent years [80–82]. Under conditions of sustained oxidative stress, free electrons on the mitochondrial electron transport chain may leak into the organelle, where it reacts with molecular oxygen to generate superoxide anions, metabolic byproducts, during respiration [83]. In addition, mitochondria are proapoptotic targets of oxidative stress factors [84]. Oxidative stress induces the instantaneous opening of permeability transition pores in the inner mitochondrial membrane and leads to the loss of the mitochondrial membrane potential; it also triggers the release of cytochrome C and apoptosis-inducing factor (AIF), which both interact with caspase-9 to form apoptotic vesicles that activate caspase-3 [85, 86]. In addition, oxidative stress can uncouple the mitochondrial electron transport chain, upregulate the expression of the proapoptotic protein Bax, and cause mitochondrial outer membrane rupture, leading to apoptosis [87, 88]. Numerous studies have shown that mitochondria-mediated oxidative stress and apoptosis are closely associated with ischemia/reperfusion injury, neurodegenerative disease, and cancer [89–92]. Therefore, the regulation of mitochondrial involvement in oxidative stress and apoptosis has also become one of the hot spots of research in recent years.

#### 4.3.4. Mechanism Research

The keyword cooccurrence timeline diagram and keyword burst analysis show that the mitochondrial biogenesis research direction in the past five years has tended to be directed to the study of disease mechanisms. For example, related studies have indicated that mitochondrial dysfunction caused by impaired mitochondrial biogenesis may be a pathological mechanism in many clinical diseases such as Parkinson's disease [93], schizophrenia [94], myocardial ischemia [95], heart failure [96, 97], insulin resistance [98], and cancer [99]. In addition, many studies have been devoted to exploring the corresponding therapeutic effects of certain regulatory interventions of mitochondrial biogenesis pathways. For example, necdin protects neurons by promoting mitochondrial biogenesis [100], LARP7 protects against heart failure by enhancing mitochondrial biogenesis [96], doxycycline reduces weight gain in early breast cancer patients by inhibiting mitochondrial biogenesis in mammary stem cells [101], and PGRN prevents diabetic nephropathy by inhibiting mitochondrial biogenesis [102]. Therefore, the development of targeted inhibitors for mitochondrial biogenesis has become a new direction for drug development in pharmaceutical companies [103–105].

Moreover, keywords analysis showed that the regulatory effect of herbal bioactive ingredients on mitochondrial biogenesis has become an important research direction in recent years. Related drug research has been carried out with the herbal pairs of *aconiti lateralis radix praeparata* and *zingiberis rhizome, resveratrol, berberine, salidroside, ginkgolic acid, quercetin, ginsenoside,* etc. [106–112]. These findings indicate that the active ingredients in natural products may be potential agents for regulating the therapeutic effect of mitochondrial biogenesis, leading to a new research direction.

Notably, studies into cellular mechanisms, particularly autophagy, have become another new research frontiers. Autophagy removes dead or dysfunctional organelles and other cytoplasmic components, recycling the components [113]. The degradation of mitochondria caused by autophagy directed specifically to dysfunctional mitochondria is called mitophagy. Mitochondrial homeostasis is jointly regulated by mitochondrial biogenesis and mitophagy to maintain the relative stability of mitochondrial quantity and quality in cells, which is very important for the normal physiological function of mitochondria [114]. Mitochondrial autophagy is a cellular evolutionary biological process that aims to remove dysfunctional or redundant mitochondria from the cell, thereby regulating the number of mitochondria to maintain a stable state of energy metabolism. Most studies on mitochondrial autophagy have been conducted to explore the biological efficacy of interventions to regulate the dynamic balance of mitochondrial autophagy and mitochondrial biosynthesis, e.g., nobiletin regulates mitochondrial autophagy and biosynthesis by activating the SIRT-1/FOXO3*α* pathway to improve hepatic ischemia-reperfusion injury [115]. The nuclear receptor Rev-erb-alpha regulates mitochondrial biosynthesis and autophagy to regulate skeletal muscle oxidative metabolism [116]. Progranulin can prevent diabetic foot cell injury by regulating jointly mitochondrial biosynthesis and autophagy [117]. All these studies suggested that maintaining mitochondrial homeostasis is important for its normal physiological function, and how to balance other mitochondrial biological processes related to mitochondrial biosynthesis to jointly maintain normal mitochondrial function deserves further study.

## 5. Limitations

Several limitations should be noticed. Firstly, only the literature included in the WoSCC database was searched and analyzed. Therefore, relevant literature included in other databases may be omitted, which may cause bias in the analysis results to a certain extent. However, the completeness of the literature collection in the WoSCC database has been widely recognized, and many bibliometric studies have been carried out based on the WoSCC database, reflecting the ability of its search results to reflect research results in certain fields. Secondly, only English papers were included in this study, and papers published in other languages were not searched or analyzed, which may have led to biased results. Finally, this research was based on titles but not keywords as qualifiers for literature retrieval, which may have led, to a certain extent, to literature on related themes not being included in the analysis; however, a title can better reflect the core content of a document, and therefore, the results of this study may better reflect the core research progress in the field of mitochondrial biogenesis than a similar study based on keywords.

## 6. Conclusion

In the past 20 years, the field of mitochondrial biogenesis has received increasing attention from researchers, and the number of related studies is increasing yearly. The United States of America and China are the main countries driving the development of this field, and developed countries in Asia and Europe have also carried out extensive research. However, collaboration between researchers needs to be strengthened. The subject areas involved in mitochondrial biogenesis are mainly biochemistry and molecular biology. Rick G Schnellmann and RC Scarpulla are the authors with the most published papers and cocitations, and their research results have accelerated the development of this field. The mitochondrial biogenesis process, transcriptional regulation mechanism, mitochondrial-mediated oxidative stress, apoptosis, and mechanistic exploration of the effect of exercise on mitochondrial biogenesis have remained long-standing research hotspots. In recent years, the mechanism and basis of mitochondrial biogenesis in disease pathogenesis have also been studied to determine the therapeutic mechanisms of interventions related to mitochondrial biogenesis, and the mechanism of interactions between mitophagy and mitochondrial biogenesis is a possible frontier direction. Based on bibliometric approach, this study provides a summary analysis of the literature about mitochondrial biosynthesis in the WOSCC database from 2002 to 2021, with the expectation that the results obtained in this study will help later researchers entering this research field to quickly grasp the research progress, research hotspots, major research teams, and possible future research directions.

## Figures and Tables

**Table 1:** The 10 countries and institutions with the highest volume of collaborative publications.

Rank	Country/region	Count	BC	Year	Rank	Institution	Count	BC	Year
1	USA	462	1.03	2002	1	Duke University	30	0.02	2002
2	People's R China	290	0.80	2008	2	Chinese Academy of Sciences	23	0.07	2008
3	South Korea	69	0.05	2010	3	University of Milan	18	0.00	2003
4	Italy	62	0.05	2008	4	York University	17	0.00	2003
5	Japan	55	0.16	2007	5	University of Arizona	17	0.00	2007
6	Spain	51	0.62	2007	6	Xi'an Jiaotong University	17	0.04	2010
7	Canada	48	0.00	2008	7	University of California, San Diego	14	0.07	2012
8	France	48	0.35	2007	8	Fudan University	14	0.03	2014
9	India	44	0.00	2009	9	The Medical University of South Carolina	14	0.00	2007
10	Germany	42	0.25	2008	10	Istituto Auxologico Italiano	13	0.00	2003

**Table 2:** The 10 authors with the most citations in the cooccurrence and cocitation analyses.

Rank	Author	Count	BC	Year	Rank	Cocited author	Count	BC	Year
1	Rick G Schnellmann	27	0.00	2010	1	Scarpulla RC	400	0.23	2002
2	Claude A Piantadosi	20	0.00	2007	2	Wu ZD	324	0.91	2002
3	Hagir B Suliman	18	0.00	2007	3	Puigserver P	284	0.42	2002
4	David A Hood	13	0.00	2006	4	Nisoli E	179	0.18	2004
5	Jiankang Liu	12	0.00	2008	5	Handschin C	163	0.22	2004
6	Enzo Nisoli	11	0.00	2006	6	Lin JD	147	0.07	2002
7	Michele O Carruba	8	0.00	2006	7	Jager S	145	0.09	2008
8	Natalie E Scholpa	8	0.00	2018	8	Canto C	143	0.35	2010
9	Alessandra Valerio	6	0.00	2010	9	St-Pierre J	134	0.00	2006
10	E Nisoli	6	0.00	2003	10	Lagouge M	103	0.09	2008

**Table 3:** The 10 most frequently cited subject categories of mitochondrial biogenesis publications.

Rank	Category	Count	BC	Year
1	Biochemistry & Molecular Biology	347	0.63	2002
2	Cell Biology	299	1.06	2002
3	Physiology	147	0.47	2002
4	Endocrinology & Metabolism	131	0.06	2002
5	Pharmacology & Pharmacy	114	0.30	2004
6	Neurosciences & Neurology	104	0.00	2006
7	Science & Technology-other topics	80	0.06	2002
8	Biophysics	64	0.00	2002
9	Research & Experimental Medicine	61	0.39	2006
10	Nutrition & Dietetics	55	0.18	2004

**Table 4:** The 10 journals with the most papers published in the mitochondrial biogenesis field.

Rank	Journal	Count	Impact factor (2020)	Quartile in category (JCR)	Cocited journal	Citation	Impact factor (2020)	Quartile in category (JCR)
1	PLOS One	38	3.240	Q2	Journal of Biological Chemistry	3775	5.157	Q2
2	Biochemical and Biophysical Research Communications	30	3.575	Q3	Proceedings of the National Academy of Sciences of the United States of America	2583	11.205	Q1
3	Journal of Biological Chemistry	30	5.157	Q2	Cell	1971	41.584	Q1
4	Mitochondrion	22	4.160	Q3	Nature	1736	49.962	Q1
5	International Journal of Molecular Sciences	21	5.924	Q1	Cell Metabolism	1268	27.287	Q1
6	Diabetes	17	9.461	Q1	Science	1239	47.728	Q1
7	FASEB Journal	17	5.192	Q2	PLOS One	1120	3.240	Q2
8	Free Radical Biology and Medicine	17	7.376	Q1	Diabetes	1076	9.461	Q1
9	Oxidative Medicine and Cellular Longevity	17	6.543	Q2	Journal of Applied Physiology	1067	3.532	Q2
10	Scientific Reports	16	4.380	Q1	Molecular and Cellular Biology	992	6.216	Q2

**Table 5:** The 10 most cited references in the cocitation analysis.

Rank	Title	Authors	Source title	Cocited frequency	Publication year
1	Transcriptional Paradigms in Mammalian Mitochondrial Biogenesis and Function [34]	Scarpulla	Physiological Reviews	83	2008
2	Transcriptional Integration of Mitochondrial Biogenesis [35]	Scarpulla et al.	Trends in Endocrinology and Metabolism	51	2012
3	Metabolic Control of Mitochondrial Biogenesis through the PGC-1 Family Regulatory Network [36]	Scarpulla	Biochimica Et Biophysica Acta-Molecular Cell Research	51	2011
4	Transcriptional Regulatory Circuits Controlling Mitochondrial Biogenesis and Function [37]	Kelly and Scarpulla	Genes & Development	43	2004
5	AMP-Activated Protein Kinase (AMPK) Action in Skeletal Muscle via Direct Phosphorylation of PGC-1 Alpha [38]	Jäger et al.	Proceedings of the National Academy of Sciences of the United States of America	43	2007
6	AMPK Regulates Energy Expenditure by Modulating NAD (+) Metabolism and SIRT1 Activity [39]	Cantó et al.	Nature	40	2009
7	Mitochondrial Biogenesis in Mammals: The Role of Endogenous Nitric Oxide [40]	Nisoli et al.	Science	35	2003
8	Suppression of Reactive Oxygen Species and Neurodegeneration by the PGC-1 Transcriptional Coactivators [41]	St-Pierre et al.	Cell	33	2006
9	Transcriptional Control of Mitochondrial Biogenesis and Function [42]	Piantadosi and Suliman	Annual Review of Physiology	33	2009
10	Resveratrol Improves Mitochondrial Function and Protects Against Metabolic Disease by Activating SIRT1 and PGC-1 Alpha [43]	Lagouge et al.	Cell	26	2006

**Table 6:** The 20 keywords in the cooccurrence frequency analysis.

Rank	Keywords	Count	BC	Rank	Keywords	Count	BC
1	Mitochondrial biogenesis	385	0.23	11	Mechanism	81	0.00
2	Skeletal muscle	269	0.40	12	Protein	75	0.04
3	Oxidative stress	259	0.38	13	Apoptosis	58	0.00
4	Gene expression	238	0.17	14	Nitric oxide	56	0.00
5	PGC-1 alpha	177	0.21	15	Transcription	52	0.00
6	Dysfunction	169	0.04	16	Coactivator	50	0.00
7	Activation	166	0.43	17	Exercise	50	0.09
8	Metabolism	164	0.13	18	Transcription factor	49	0.10
9	Activated protein kinase	94	0.77	19	Cell	48	0.00
10	Insulin resistance	83	0.17	20	Disease	44	0.00

## Data Availability

The original materials related to this study are provided in the main text or supplementary materials. Further inquiries can be directed to the corresponding author by email.
